# Estimation of Air Damping in Out-of-Plane Comb-Drive Actuators

**DOI:** 10.3390/mi10040263

**Published:** 2019-04-19

**Authors:** Ramin Mirzazadeh, Stefano Mariani

**Affiliations:** Department of Civil and Environmental Engineering, Politecnico di Milano, Piazza L. da Vinci 32, 20133 Milano, Italy; ramin.mirzazadeh@polimi.it

**Keywords:** resonant micro-electromechanical-systems (MEMS), micro-mirrors, out-of-plane comb actuation, fluid damping, analytical solution, FE analysis

## Abstract

The development of new compliant resonant microsystems and the trend towards further miniaturization have recently raised the issue of the accuracy and reliability of computational tools for the estimation of fluid damping. Focusing on electrostatically actuated torsional micro-mirrors, a major dissipation contribution is linked to the constrained flow of air at comb fingers. In the case of large tilting angles of the mirror plate, within a period of oscillation the geometry of the air domain at comb-drives gets largely distorted, and the dissipation mechanism is thereby affected. In this communication, we provide an appraisal of simple analytical solutions to estimate the dissipation in the ideal case of air flow between infinite plates, at atmospheric pressure. The results of numerical simulations are also reported to assess the effect on damping of the finite size of actual geometries.

## 1. Introduction

Starting from the late 1990s, the fields of application of micro-electromechanical-systems (MEMS) have been progressively expanding [1,2], leading to state-of-the-art devices like accelerometers and gyroscopes [3], gas sensors [4], bio-MEMS [5], and micro-opto-electromechanical-systems (MOEMS) [6], which are now available at low cost.

For a resonating system, energy dissipation plays an important role in modulating its response; such dissipation is customarily measured via the quality factor of the structure. High-resonance frequencies, achievable with miniaturization, and low dissipation can provide a better system response; nevertheless, moving from the macro-scale to the micro-scale, surface effects such as the viscous and electrostatic effects become dominant and need to be accurately quantified.

Comb-actuated resonant micro-mirrors (see, e.g., [7,8]) are a good example of systems designed to work at high frequencies to attain high scanning resolutions, despite the considerable dissipation arising from the interaction of the resonating structure with the surrounding air at atmospheric pressure. Besides the need to interact with the optical beams, these devices are preferred to operate at atmospheric pressure since fluid damping can avoid the risks of excessive dynamic amplifications and sharp frequency responses, and so ultimately of possible structural failures. The fluid behavior and, consequently, the dissipation induced on such devices might not be the same as those at the macroscale, due to rarefaction effects [2,9,10,11]. By considering the steady-state flow of a thin gas film between oscillating parallel plates, boundary conditions (BCs) should be modified to consider a finite slip at the fluid-structure interface. A so-called first-order finite slip model was reported in [12] to account rather accurately for the rarefaction effects at large values of the Knudsen number Kn, which is defined in the considered case as the ratio between the mean free path of air molecules and the width of the film. Even for geometries and working pressures leading to values of Knudsen number approaching Kn=1, such slip BCs give rise to errors in terms of damping smaller than 10%.

For micro-mirrors actuated by comb finger arrays, the damping observed between two closely placed plates having a relative sliding motion is of major importance, due to the small gap between rotors and stators for actuation purposes. This type of fluid damping at the microscale was already investigated, e.g., in [13,14], by running experiments on parallel plates and modelling the relevant dissipation mechanisms; an empirical formula was also proposed in [15] for specific geometries. In [16,17], the Stokes equations were numerically solved for three-dimensional geometries via a fast Fourier transform (FFT) boundary element method, considering both the standard no-slip, or stick BCs and the rarefaction effects (see also [18]). These studies and those based on Navier–Stokes modeling approaches (see, e.g. [19,20]) focused on damping in the case of relatively small amplitudes of oscillations. For scanning reasons, micro-mirrors are instead designed to have large tilting angles (in the order of 15° or even more), which alter the fluid flow in comparison to the small oscillation case: the flow turns out to be far more complex and needs to be modelled numerically.

In order to provide an estimation for the real solution in terms of damping, namely, in terms of the ratio between the energy loss and the maximum energy stored within a period of oscillation of the mechanical system, a simplified analytical model is here discussed and validated against finite element simulations. Referring to the free vibrations of a (linear) system, in the ideal case of no damping the energy conservation law states that there must be a continuous switch between a purely internal or elastic contribution (when, e.g., the torsional velocity of the considered micro-mirror is zero) and a purely kinetic one (when, e.g., the torsional angle is zero). In the presence of damping, the sum of the elastic and kinetic contributions decays in time; in the viscous case, an effective damping parameter d quantifies such decay, as induced by the surrounding air on the motion of the movable structure. This parameter can be combined with the effective mass and stiffness of the structure, to provide the aforementioned quality factor.

**Figure 1:**
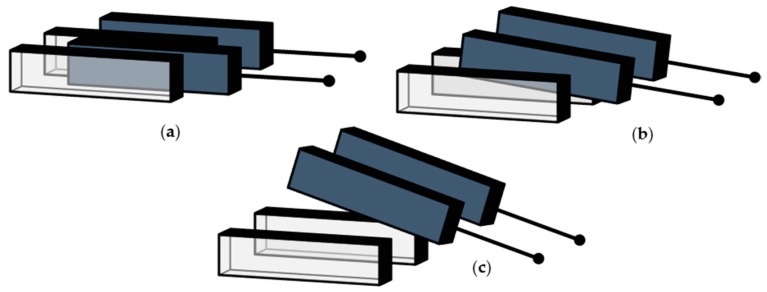
Schematics of stator/rotor configurations at comb fingers, during the torsional motion of a micro-mirror: (**a**) engaged, (**b**) partially engaged, and (**c**) disengaged phases.

Moving from already reported analyses, a solution is presented in Section 2 for the constrained flow of a fluid in a narrow gap between two parallel infinite plates. Two different solutions are detailed: a so-called disengaged one, when both plates are moving in-phase; and a so-called engaged one, when only one plate moves. Keeping aside the possible effects of finite slip, in the former case the velocity of the fluid is imposed equal at the boundaries with both the lateral surfaces; in the latter case, the velocity is instead non-zero at the boundary with one lateral surface only. These two solutions have been considered representative of the flow conditions occurring at the comb fingers of a compliant torsional micro-mirror, as schematically depicted in Figure 1: terms engaged and disengaged, respectively, refer to the solution corresponding to the torque angle being zero or maximum. In the engaged case (Figure 1a), rotors and stators are perfectly inter-digitated; in the disengaged case (Figure 1c), the local out-of-plane motion of the rotor does not allow the two sets of surfaces to directly interact. All the other conditions between these two extrema provide a partial engagement of the surfaces; accordingly, solutions for the studied cases provide a kind of bilateral bounding on any possible real configuration occurring during the torsional vibration of the moving structure. The estimated values of the damping coefficient in the two conditions are discussed in Section 3, at varying frequency of the oscillations; further to that, the effect of a finite size of the plates is evaluated numerically, so that charts can be made available for the estimation of dissipation in any geometry. Some concluding remarks are gathered in Section 4.

## 2. Theoretical Analysis of the Constrained Shear Flow

Let two flat surfaces, bounding a gas film, be separated by a gap H and move in a direction parallel to each other. Referring to the configurations depicted in Figure 1, this geometry is relevant to a cross section of the comb-drives; the torsional motion of the micro-mirror induces a sliding between the lateral surfaces of rotor and stator, measured by the considered motion along the x-axis.

Starting from the Navier–Stokes equations, by neglecting the inertial forces and assuming that a quasi-static flow forms between the plates, a frequency-independent solution can be obtained. Here, we are instead interested in a vibrating structure, and so a time- and frequency-dependent velocity field must be computed for the fluid film. Some assumptions and restrictions are introduced to deal with the problem analytically: (i) the amplitude of oscillations is small in comparison with H; (ii) the thickness and length of the plates are large with respect to H; (iii) the velocity of the surfaces is low enough to prevent heating of the fluid; (iv) surfaces are ideally smooth at the molecular level, so that the angles of incidence and reflection of gas molecules colliding with them are identical, and the relevant tangential momentum does not change. The effects of assumption (ii) will be specifically assessed in Section 3, via numerical simulations dealing with finite plate geometries; assumption (iii) is instead introduced to prevent any additional thermal dissipation effect. Due to assumption (i), a switch between the engaged and disengaged configurations cannot be dealt with within a single solution; we therefore study the two different cases separately, in order to provide an estimation of the dissipation valid for small perturbations within the two regimes. The analytical model is thus incapable to provide an assessment of dissipation coping with the structural vibrations within a complete cycle.

**Figure 2:**
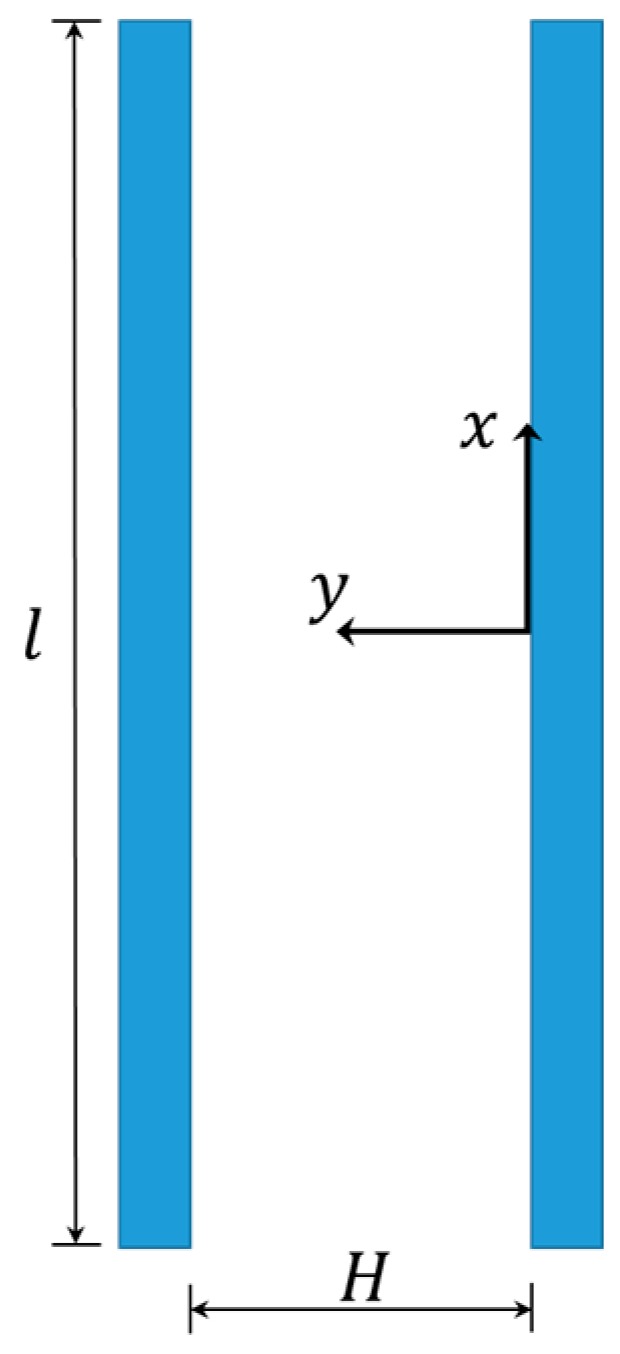
Lateral view of the parallel plates and of the considered fluid film in between: notation.

In the reference frame depicted in Figure 2, with the x-axis aligned with the motion of the fluid and the y-axis perpendicular to the moving surface(s), the dynamics of the gas between the plates are modeled with the following one-dimensional diffusion equation [20,21]:

(1) $$\frac{\partial u\left(y\right)}{\partial t}=\nu \frac{\partial^{2}u\left(y\right)}{\partial y^{2}}$$

where u is the fluid velocity, function of the y coordinate only in the present ideal case; t is time; and ν=μ/ρ is the kinematic viscosity of the fluid, μ its coefficient of dynamic viscosity, and ρ its density. In Equation (1), it has been assumed that the velocity of the oscillating plate(s) and the frequency of oscillations are such that air can be treated as incompressible, see [22,23]; recall also that a limitation on the value of the velocity u is enforced by assumption (iii) [12].

Equation (1) is solved in the case of a steady-state sinusoidal excitation at one or both borders of the film, featuring an amplitude $\bar{u}$ and a circular frequency ω. The solution, therefore, can be written as follows:

(2) $$u\left(y\right)=C_{1}\sinh\left(qy\right)+C_{2}\cos h\left(qy\right)$$

where $q=\sqrt{j\omega /\nu }$ is the complex frequency variable and j is the imaginary unit. Constants $C_{1}$ and $C_{2}$ vary, depending on the BCs at the borders; solutions are therefore distinguished next, to deal with the disengaged and engaged cases separately.

The shear stress τ acting on the rotor (moving) wall is given by

(3) $$\tau =-\mu {\frac{\partial u\left(y\right)}{\partial y}|}_{y=H}$$

where the minus sign depends on the relative orientation of surface motion and velocity gradient, and states that the shear stress actually provides the source of dissipation. The quantity

(4) $$\xi ={\frac{\tau }{\bar{u}}}_{}$$

that is, the ratio between the shear stress acting on the rotor surface and the imposed surface velocity, is the damping admittance ξ. For a periodic steady-state solution, the real part of this complex quantity yields the actual damping coefficient per unit area.

### 2.1. Disengaged Solution: Two Moving Surfaces 

We investigate first the flow between two plates oscillating in-phase with the same velocity $\bar{u}$. In case of flow at low frequencies, the velocity u turns out to be uniform all over the domain 0≤y≤H and equal to the imposed one at the boundaries. As the frequency increases, the inertial forces assume importance and the local velocity becomes dependent on the position too, showing a Poiseuille-type distribution [24]. 

For stick BCs, namely, for $u\left(0\right)=u\left(H\right)=\bar{u}$, the velocity distribution in the gap turns out to be

(5) $$u\left(y\right)=\bar{u}\left[\frac{1-\cosh\left(qH\right)}{\sinh\left(qH\right)}\sinh\left(qy\right)+\cos h\left(qy\right)\right]$$

For slip BCs, namely, for $u\left(0\right)=\bar{u}+\lambda {\frac{\partial u\left(y\right)}{\partial y}|}_{y=0}$ and $u\left(H\right)=\bar{u}-\lambda {\frac{\partial u\left(y\right)}{\partial y}|}_{y=H}$, the velocity gradient $\frac{\partial u\left(y\right)}{\partial y}$ at the two surfaces and the mean free path of gas molecules λ affect the solution. Out of the considered ideal specular reflection condition, the solution also depends on the tangential momentum accommodation coefficient (see, e.g., [25]). The relevance of slip BCs is determined by the Knudsen number Kn, or by the ratio between the aforementioned mean free path λ and the characteristic physical length-scale H of the problem: when the two variables become comparable, stick BCs lead to an overestimation of damping, and a correction of continuum methods via slip BCs looks necessary. For this type of BCs, the velocity distribution thus becomes

(6) $$u\left(y\right)=\bar{u}\left[K^{\prime }\sinh\left(qy\right)+\left(1-\lambda qK^{\prime }\right)\cos h\left(qy\right)\right]$$

where

(7) $$K^{\prime }=\frac{1-\cosh\left(qH\right)-\lambda q\sin h\left(qH\right)}{\sinh\left(qH\right)+2\lambda q\cos h\left(qH\right)+\lambda^{2}q^{2}\sinh\left(qH\right)}$$

Flows are studied for H=3 and 12 μm; these values of the gap have been chosen as being representative of the micro-mirror geometry considered in [7,8]. As already discussed, micro-mirrors typically work at ambient pressure, so that λ=69 nm, $\mu =18.3\times 10^{-6}\;\mathrm{kg}/m$s, $\rho =1.185\;\mathrm{kg}/m^{3}$.

**Figure 3:**
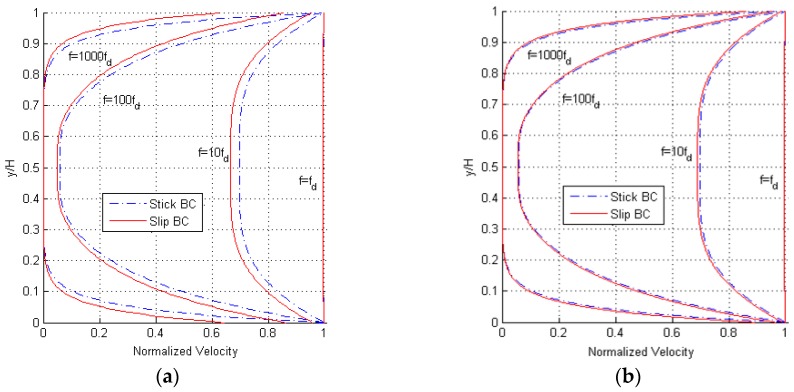
Disengaged solution, (**a**) H=3 μm and (**b**) H=12 μm: maximum amplitude of the velocity profile at varying frequency f of oscillation of the two plates, for both stick and slip boundary conditions (BCs).

Results are provided in Figure 3, in terms of the profile of the maximum amplitude of the velocity between the two oscillating surfaces. Having defined the corner frequency as $f_{d}=\nu /\left(2\pi H^{2}\right)$ [12], for which |qH|=1, it can be seen that for frequencies smaller than $f_{d}$ the inertial forces do not have any effect on the flow, and the fluid moves uniformly with the same velocity of the bounding surfaces. For frequencies larger than $f_{d}$, the inertial forces change the velocity profile to approach one mimicking a pressure-driven Poiseuille flow. The ratio $f/f_{d}$, also termed normalized frequency in the following, can therefore be considered as an implicit measure of the Reynolds number of the solution. At even higher frequencies, inertial forces get dominant and only a small part of the gas film, close to the moving surfaces, moves along: this portion of the gap gets narrower as the frequency increases. While the shape of the profiles is not affected by the value of H, it can be seen that the difference between the solutions relevant to stick and slip BCs becomes negligible for H=12 μm. It must be anyway kept in mind that, by increasing the gap from H=3 μm to H=12 μm, the corner frequency decreases from $f_{d}=323,615\;\mathrm{Hz}$ to $f_{d}=20,225\;\mathrm{Hz}$; this means that the inertial forces provide consistent effects on the solution for lower frequencies, in case of larger gaps.

**Figure 4:**
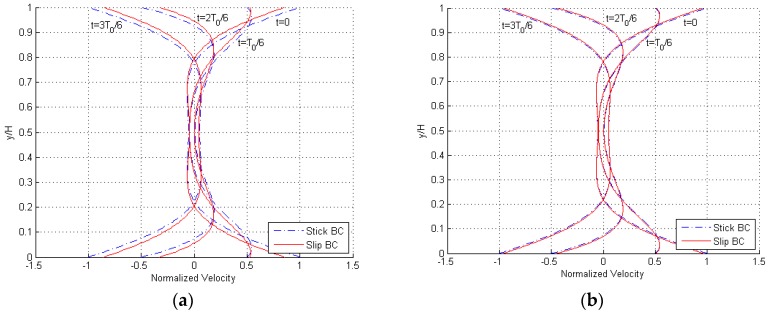
Disengaged solution, (**a**) H=3 μm and (**b**) H=12 μm: snapshots of the velocity profile during a single cycle of oscillation of the two plates, for $f=100f_{d}$ and both stick and slip BCs.

In Figure 4, snapshots of the velocity profile during a cycle of the steady-state flow are depicted; these curves are shown for a frequency $f=100f_{d}$, to testify the effects of inertia. Once more, it can be seen that the effects of slip BCs become negligible for H=12 μm. The overall shape of the profile is again unaffected by H, as the solution is scale-independent in this dimensionless representation.

### 2.2. Engaged Solution: One Moving Surface 

We investigate now the flow between the same two plates, assuming the surface at y=0 to be fixed and the one at y=H to move as before with an assigned velocity $\bar{u}$. In the case of stick BCs, namely, for u(0)=0, $u\left(H\right)=\bar{u}$, the velocity distribution reads as follows:

(8) $$u\left(y\right)=\bar{u}\frac{\sinh\left(qy\right)}{\sinh\left(qH\right)}$$

For slip BCs, namely, for $u\left(0\right)=\lambda {\frac{\partial u\left(y\right)}{\partial y}|}_{y=0}$ and $u\left(H\right)=\bar{u}-\lambda {\frac{\partial u\left(y\right)}{\partial y}|}_{y=H}$, the velocity profile results instead are as follows [12]:

(9) $$u\left(y\right)=\bar{u}\frac{\sinh\left(qy\right)+q\lambda \cos h\left(qy\right)}{\left(1+q^{2}\lambda^{2}\right)\sinh\left(qH\right)+2q\lambda \cos h\left(qH\right)}$$

**Figure 5:**
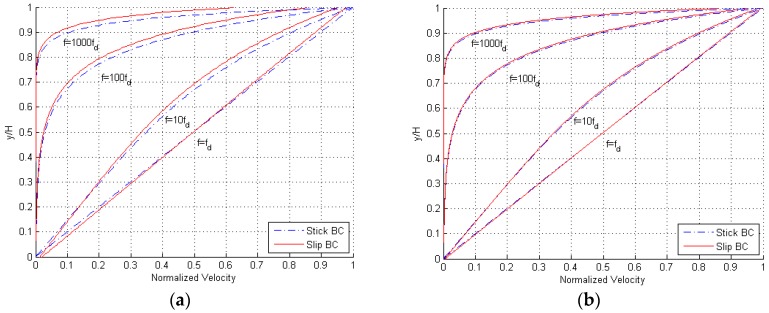
Engaged solution, (**a**) H=3 μm and (**b**) H=12 μm: maximum amplitude of the velocity profile at varying frequency f of oscillation of the moving plate at y=H, for both stick and slip BCs.

**Figure 6:**
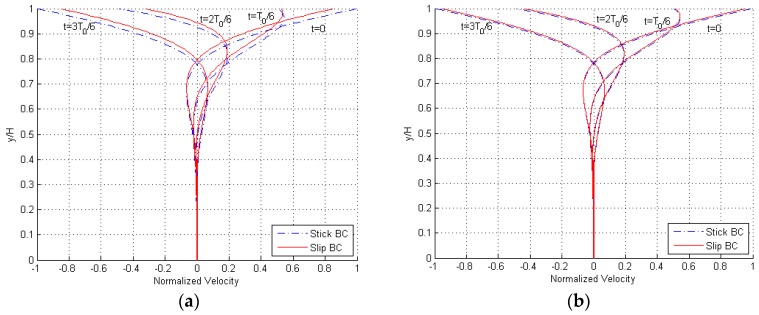
Engaged solution, (**a**) H=3 μm and (**b**) H=12 μm: snapshots of the velocity profile during a single cycle of oscillation of the plate at y=H, for $f=100f_{d}$ and both stick and slip BCs.

Results are reported in Figure 5 in terms of the profile of the maximum amplitude of the velocity at varying frequency of oscillations of the plate at y=H, for $f\geq f_{d}$ and both stick and slip BCs. Some snapshots within a single cycle of the steady-state solution are depicted in Figure 6, for $f=100f_{d}$. Profiles are again provided for H=3 and 12 μm, and, in accordance with the disengaged solution, it can be seen that by increasing the gap, the effects of the velocity slip at the boundaries get smaller, becoming almost negligible for the larger value. The only feature to notice in the graphs, at variance with the former solution, can be observed at y=0: for frequencies much larger than $f_{d}$, the gradient of the solution u(y) close to the fixed surface becomes zero and so the velocity slip at the boundary becomes null as well. In Figure 6, the solution is thus displayed to be always zero at the bottom of the plots, independently of the time instant within the cycle.

## 3. Estimation of the Damping Coefficient

**Figure 7:**
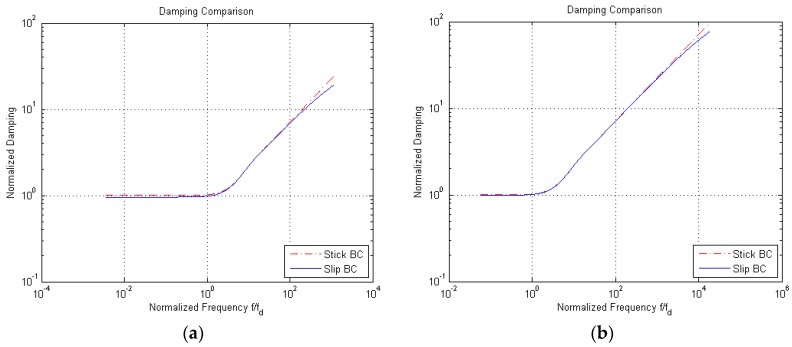
Engaged solution, (**a**) H=3 μm and (**b**) H=12 μm: normalized damping at varying frequency f, for both stick and slip BCs.

The goal of the analytical model is, as said, an estimation of damping in the considered cases of shear flow. If the real part of the admittance ξ, which is already computed per unit area via Equation (4), is normalized by μ/H, the effects of the oscillation frequency on the damping are as shown in Figure 7 for the engaged case; in these plots, as before, we have considered the two values H=3 and 12 μm for the gap, and both stick and slip BCs. By means of the handled nondimensionalization for the damping coefficient, a direct comparison is provided with the dissipation induced by the Couette flow with no-slip BCs, a linear profile of the fluid velocity along the y-axis, and a constant gradient equal to $\bar{u}/H$. For brevity, results are not reported for the disengaged case, but a comparison between the solutions obtained with the two types of BCs is shown next.

In Figure 7, it can be seen that damping at frequencies smaller than $f_{d}$ is constant, due to the null effect of the fluid inertia on the shape of the velocity profile. The values of the normalized damping for slip BCs are slightly smaller than those relevant to stick BCs, for all the scanned frequencies; this outcome is obviously linked to the lag in the solution induced by the slip at the boundaries. Additionally, this effect gets almost negligible in the case H=12 μm, except for very high-frequency values, above $1000f_{d}$, for which the two curves tend to diverge.

**Figure 8:**
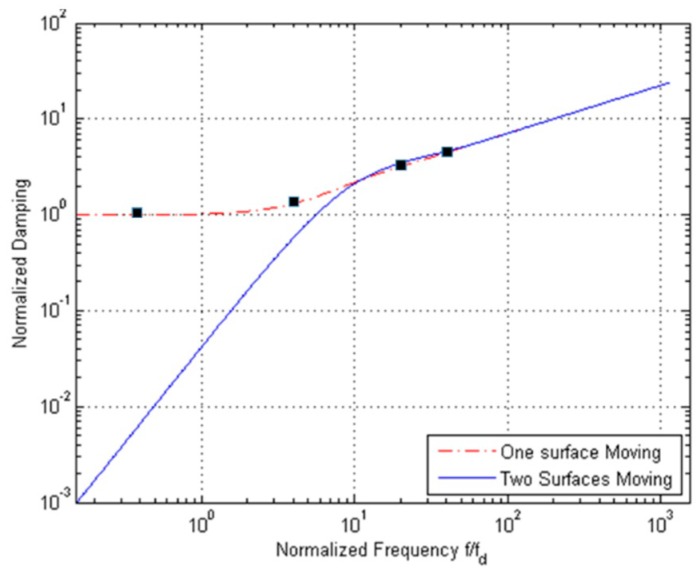
H=3 μm, stick BCs: comparison between the values of normalized damping for engaged and disengaged solutions.

Due to the small difference between the solutions relevant to the two types of BCs over a broad range of frequencies, we refer now to the stick BCs only. Figure 8 shows the normalized damping values for the engaged and disengaged solutions (respectively, denoted in the graph as one surface and two surfaces moving), as a function of the vibration frequency f. Damping for the disengaged case is negligible at low frequencies; by increasing the frequency, damping values for the two cases get closer and closer and then show the same trend. This outcome is due to the fact that at high frequencies only a portion of the air film moves with the wall(s), and the solution in terms of the damping coefficient thus becomes independent of the type of BCs.

**Figure 9:**
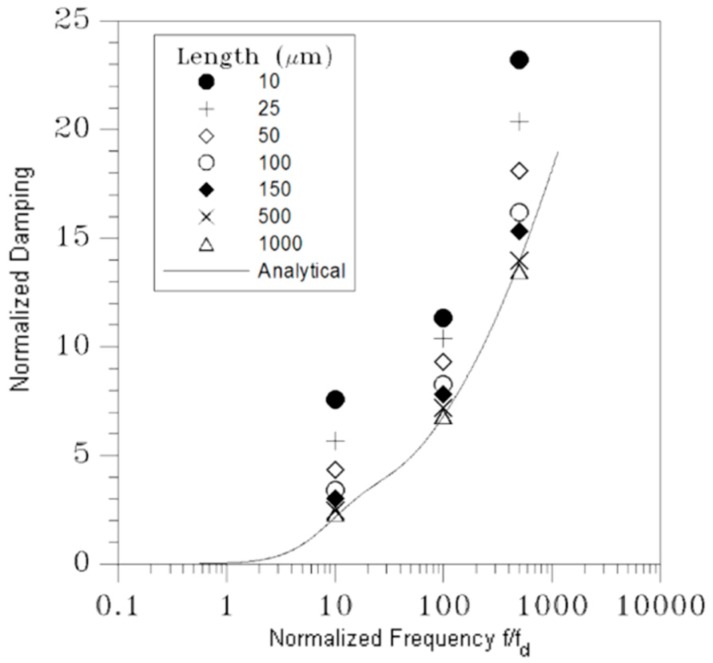
H=3 μm, disengaged solution, stick BCs: effect of surface length on the normalized damping value.

In Figure 8, the black square symbols refer to the data reported in [12]: to validate the solution, results of numerical analyses were reported for the engaged case and a geometry characterized by l/H=1000, allowing for finite slips at the boundaries. Having thereby minimized the fringe effects and set the corner frequency as $f_{d}=2.5$ MHz, the numerical results turn out to be in good agreement with the analytical solution, featuring a discrepancy of a few % only in the whole inspected frequency range. To also assess the accuracy of the solution in the disengaged case, and the effects of fringe flow on the damping behavior of real geometries with shorter plates, numerical analyses have been carried out. The solution reported in Figure 9 has been computed with ANSYS CFX (v13.0, Ansys Inc., Canonsburg, PA, USA) [26] for frequencies equal to $10f_{d}$, $100f_{d}$, and $500f_{d}$, at a varying length of the two plates.

The finite-length numerical model has been setup with the fluid between the two plates and also surrounding their front and rear surfaces, to catch the aforementioned fringe effects during a cycle of the assigned sinusoidal motion. The width of the additional lateral fluid regions has been selected to attain a width-independent solution, and thus avoid any numerical artifact. Open BCs have been adopted on the two outer sides of the model, to guarantee uniform in and out flows. The space discretization of the fluid domain, for any frequency, has been checked to assure mesh-independent results. To cross-validate the analytical and numerical approaches, a relatively long surface featuring l=1000μm has been first adopted: results in Figure 9 show that the analytical solution is perfectly matched up to frequencies on the order of $100f_{d}$; the numerically computed damping is instead slightly smaller than the analytical one at $500f_{d}$, but still within the accuracy margin already reported for the engaged case. Next, a series of simulations has been carried out at varying plate length, or gap over length ratio, in the range H/l=0.003−0.3. The computed values of the damping coefficient obviously turn out to be larger for the shorter plates, since air flow is affected more by the fringe flow. Results relevant to H=3 μm show that, for H/l=0.03, the damping is increased by 57%, 21%, and 16% at frequencies $10f_{d}$, $100f_{d}$, and $500f_{d}$, respectively. Such an increase with respect to the shear-dependent analytical baseline has to be linked to the pressure-dependent effects related to the front and rear surfaces of the plates, which are not included in the analytical solution; the interplay of these effects was already assessed numerically in, e.g., [22,23]. 

The reported variation of the damping coefficient turns out to be proportional to the ratio between the gap and the surface length, as for H/l=0.12 it grows up to 163%, 53%, and 49% at the same frequencies. To cope with these variations of damping away from the analytical solution, in [12] an empirical law was proposed for the engaged case, to define an effective gap width in the form of $H^{*}=H^{*}\left(H,H/l\right)$. This solution may turn out to be case-dependent, since the actual three-dimensional geometry of the moving structure and its kinematics should be fully accounted for in $H^{*}$. Alternatively, compact modeling approaches resting on an equivalent electric circuit for the fluid film can be formulated as in [12]; again for the engaged case, this perspective was shown to provide very accurate solutions for frequencies below the corner one, while for higher values some fluctuations were reported for the frequency-dependent damping. In this communication, we do not aim to discuss methods to cope with case-dependent geometries; instead, analytical results related to the dependence of damping on the resonance frequency of the movable structure are intended to provide a simple estimation to help in the design of new devices. More accurate investigations, even supported by charts accounting for the finite geometry of the moving objects and built upon data like those reported in Figure 9, can then allow one to tune the damping of the whole structure and better fit the performances required for specific applications.

## 4. Conclusions

In this paper, we have provided an appraisal of analytical solutions to estimate energy dissipation in the case of shear flow within a thin air film bounded by two infinite plates. The values of the damping coefficient, measuring energy dissipation in the considered dynamic solution, have been compared with those obtained with finite element simulations, run to assess the effects of a finite length of the moving plate(s) and, thus, of fringe flow. Through these analyses, the amplification of the analytical damping coefficient has been discussed, as a function of the vibration frequency and of the ratio between the plate length and the gap between the plates.

For micro-mirrors characterized by a compliant movable structure due to scanning reasons, we have discussed how the fluid damping can be difficult to compute at comb-drives: as large values of the oscillation angle induce a finite change of the film configuration during each cycle of actuation, mesh updating procedures are necessary in cumbersome finite element simulations [7]. To provide a guideline for the design of new devices, at least for what concerns the dissipation at the said comb-drives, the analytical solutions here detailed can be considered as representative of states close to a null rotation of the micro-mirror (termed engaged case) and close to the maximum tilting angle (termed disengaged case). The analytical solutions relevant to infinitely long films can then be corrected through a set of numerical simulations giving information on how the front and rear sides of each finger affect the dissipation. Referring to the operational conditions and to a target layout, such corrective terms have to be computed only for specific values of the frequency of oscillations.

In the proposed analysis, the geometry of the fluid domain has been considered deterministically known. Stochastic effects induced by micro-fabrication, see, e.g., [27,28,29,30,31], have been therefore disregarded, though they may modify the solution by affecting the gap between rotor and stator. Within the current analytical frame, by partially allowing for the finite geometry of comb-drives, stochastic effects induced by microfabrication can be accounted for rather easily, to provide confidence intervals for the damping coefficient. The mentioned intervals would help in the design of new devices, especially with an eye towards further miniaturization and towards the development of high-frequency piezoelectric resonators, featuring performance indices to be finally compared with case-specific experimental data.
